# Dietary Medium-Chain Triglyceride Decanoate Affects Glucose Homeostasis Through GPR84-Mediated GLP-1 Secretion in Mice

**DOI:** 10.3389/fnut.2022.848450

**Published:** 2022-03-24

**Authors:** Hazuki Nonaka, Ryuji Ohue-Kitano, Yuki Masujima, Miki Igarashi, Ikuo Kimura

**Affiliations:** ^1^Department of Applied Biological Science, Graduate School of Agriculture, Tokyo University of Agriculture and Technology, Fuchu-shi, Japan; ^2^Laboratory of Molecular Neurobiology, Graduate School of Biostudies, Kyoto University, Kyoto, Japan; ^3^Department of Molecular Endocrinology, Graduate School of Pharmaceutical Sciences, Kyoto University, Kyoto, Japan

**Keywords:** capric acids, GPR84, gut hormone, GLP-1, obesity, decanoic acid

## Abstract

**Background:**

Dietary triglycerides are an important energy source; however, their excess intake causes metabolic diseases such as obesity and type 2 diabetes. Medium-chain triglycerides (MCTs) as triglyceride forms of medium-chain fatty acids (MCFAs) are applied to meet the energy demands of athletes, the elderly, and people with stunted growth, because MCFAs are efficiently converted into energy for immediate utilization by the organs and do not accumulate as fat. Although the intake of each MCT type (octanoate; C8:0, decanoate; C10:0, and dodecanoate; C12:0) exhibits beneficial metabolic effects, individual functional differences remain unclear.

**Methods:**

MCTs or MCFAs were administrated to male GPR84-deficient mice with a C57BL/6J background and mouse enteroendocrine cell line STC-1, and the effects on glucose homeostasis and gut hormone GLP-1 secretion were evaluated.

**Results:**

C10:0 intake improves glucose metabolism through the MCFA receptor GPR84-mediated GLP-1 secretion. Each MCT intake showed resistance to obesity and improved metabolic parameters compared with lard intake. Moreover, oral administration of MCTs enhanced glucose tolerance, especially C10:0 administration, which sufficiently increased plasma GLP-1 levels. Additionally, C10:0 stimulation promoted GLP-1 secretion via GPR84 in STC-1, enhanced glucose tolerance through GPR84-mediated GLP-1 secretion, and showed resistance to high-fat diet (HFD)-induced obesity in mice.

**Conclusions:**

Dietary MCT (C10:0) intake efficiently may protect against obesity and improve insulin resistance via GLP-1 secretion.

## Introduction

Medium-chain triglycerides (MCTs) consisting of three medium-chain fatty acids [MCFAs; octanoate (C8:0), decanoate (C10:0), and dodecanoate (C12:0)] attached to a glycerol molecule are a unique form of dietary fat. They have various health benefits and are involved in metabolic regulation and brain function (1–3). MCT-derived MCFAs are rapidly metabolized as fuel in the liver, because MCFAs with chain lengths shorter than those of long-chain fatty acids (LCFAs) can pass through the portal vein after absorption in the intestine because of their water solubility (4, 5). Hence, instead of accumulating as fat, MCFAs are efficiently converted into energy for immediate organ utilization. Additionally, MCT intake also increases the energy source by the rapid formation of ketone bodies, because an excess of acetyl-CoA is produced by metabolizing MCFAs in the liver (4, 6). Thus, MCTs can be applied to people with high energy demands, such as athletes for enhancing their physical performance, the elderly who experience a decline in energy production due to aging, subjects undergoing surgery, and persons with stunted growth (7–10).

Fatty acids act not only as energy sources, but also as signal molecules via free fatty acid receptors (FFARs) known as G-protein coupled receptors (GPRs) (1). GPR41 and GPR43 are receptors for short-chain fatty acids (SCFAs), and GPR40 and GPR120 are receptors for long-chain fatty acids (1). MCFAs also act as ligands for GPR84 and GPR40 (1, 11). GPR84 is a specific receptor for MCFAs and is coupled with the pertussis toxin-sensitive Gi/o protein (1, 11). GPR84 is mainly expressed in the bone marrow and metabolic tissues, and some studies have indicated that GPR84 has important metabolic functions (1, 12, 13). However, the molecular mechanism underlying the association between dietary MCTs and GPR84 remains unclear.

Both dietary long-chain triglycerides (LCTs)-derived LCFAs and gut microbial SCFAs affect energy metabolism and glucose homeostasis through the secretion of gut hormones, such as glucagon-like peptide-1 (GLP-1), via GPR40, GPR120, GPR41, and GPR43 (1). GLP-1, an incretin released from enteroendocrine L cells, contributes to the energy metabolism by attenuating the postprandial glycemic response via insulin secretion from pancreatic beta-cells and suppressing appetite via the central nervous system (14, 15). However, the relationship between GLP-1 secretion and dietary MCT-derived MCFA-mediated GPR84 action remains unclear. In this study, we investigated the influence of dietary MCTs-derived MCFAs, especially decanoate, by using GPR84-deficient mice and a high-fat diet (HFD)-induced obese mouse model.

## Materials and Methods

### Animals and Diet

Male C57BL/6J mice were purchased from Japan SLC (Shizuoka, Japan), housed in a conventional animal room at 24°C, and maintained under a 12 h light/dark cycle. *Gpr84*^−/−^ mice with a C57BL/6J background were generated using the CRISPR/Cas9 system. Mice were acclimated to the CLEA Rodent Diet (CE-2, CLEA Japan, Inc., Tokyo, Japan) for 1 week prior to treatment. Seven-week-old mice were placed on an MCT diet or modified D12492 diet (60% kcal fat, Research Diets Inc., New Brunswick, NJ, USA) for 5 weeks. These diets were formulated as either lard or MCT (C8:0 triglyceride, C10:0 triglyceride, or C12:0 triglyceride, Nisshin OilliO Group, Ltd., Tokyo, Japan), or based on the D12492 diet (Research Diets Inc.) supplemented with or without 5% decanoate (C10:0, FUJIFILM Wako Pure Chemical Corporation, Osaka, Japan). The composition of the diets is provided in Supplementary Tables 1, 2. Body weight was measured once per week for the duration of the experiment. All mice were sacrificed under deep isoflurane induced anesthesia. All experimental procedures involving mice were performed according to protocols approved by the Committee on the Ethics of Animal Experiments of the Kyoto University Animal Experimentation Committee (Lif-K21020) and the Tokyo University of Agriculture and Technology (permit number: 28–87). All efforts were made to minimize suffering.

### Biochemical Analyses

Blood glucose levels were assessed using a portable glucometer (OneTouch® Ultra®, LifeScan, Milpitas, CA, USA). The concentrations of plasma triglycerides (TG) (LabAssay™ Triglyceride, FUJIFILM Wako Pure Chemical Corporation), non-esterified fatty acids (NEFAs) (LabAssay™ NEFA, FUJIFILM Wako Pure Chemical Corporation), and cholesterol (LabAssay™ Cholesterol, FUJIFILM Wako Pure Chemical Corporation), were measured according to the manufacturer's instructions. Plasma insulin [(Insulin enzyme-linked immunosorbent assay (ELISA) KIT (RTU), Shibayagi, Gunma, Japan] and GLP-1 [glucagon-like peptide-1 (Active) ELISA, Merck Millipore, Darmstadt, Germany] levels were determined using ELISA as described previously (16). For plasma GLP-1 measurement, the samples were treated with a dipeptidyl peptidase IV (DPP-IV) inhibitor (Merck Millipore) to prevent the degradation of active GLP-1.

### MCFA Measurement

The MCFA levels in the plasma and intestinal samples were determined following a previously described protocol with modifications (17). Briefly, the samples containing an internal control (C19:0) were homogenized in methanol and mixed with chloroform and water to extract the lipids. The samples were centrifuged at 2,000 × *g* at 17 °C for 10 min. The supernatant containing MCFAs was collected and dried. The samples were resuspended in chloroform:methanol (1:3, v/v) and subjected to liquid chromatography with tandem mass spectrometry (LC-MS/MS) analysis using an ultra-performance LC system (UPLC, Waters, Milford, MA, USA) equipped with an Acquity UPLC system coupled to a Waters Xevo TQD mass spectrometer (Waters). The samples were separated on an ACQUITY UPLC BEH C18 column (2.1 × 150 mm, 1.7 μm; Waters) using a methanol gradient in 10 mM ammonium formate aqueous solution.

### Glucose Tolerance Tests

For the oral glucose tolerance test (OGTT), mice were allowed to fast for 16 h, and administered MCT (C8:0 triglyceride, C10:0 triglyceride, or C12:0 triglyceride; 2.4 g/kg body weight) or decanoate (C10:0; 1 g/kg body weight) in 0.5% carboxymethylcellulose through oral gavage. At 1 h post-administration (MCT administration) or 2 h post-administration (MCFA administration), mice were orally administered glucose (2.5 mg/g body weight). The plasma glucose concentration was monitored before injection and at 15, 30, 60, 90, and 120 min post-injection.

### RNA Extraction and Quantitative Reverse Transcription-Polymerase Chain Reaction

Total RNA was extracted using the RNeasy Mini Kit (Qiagen, Hilden, Germany) and RNAiso Plus (Takara, Shiga, Japan). cDNA was transcribed using total RNA as the template with Moloney murine leukemia virus reverse transcriptase (Invitrogen, CA, USA). qRT-PCR analysis was performed using SYBR Premix Ex Taq II (Takara) and the StepOne™ real-time PCR system (Applied Biosystems, CA, USA), as described previously (18, 19). The sequences of the primers were as follows: *Gpr84*, 5′-AGGTGACCCGTATGTGCTTC-3′ (forward) and 5′-AGATGATGGGATTGATCACAGGAG-3′ (reverse); *18S*, 5′-ACGCTGAGCCAGTCAGTGTA-3′ (forward) and 5′-CTTAGAGGGACAAGTGGCG-3′ (reverse).

### STC-1 Cell Culture

STC-1 cells (mouse enteroendocrine cell line; ATCC) were cultured in Dulbecco's modified eagle medium (DMEM; Sigma, St. Louis, MO, USA) containing 1% penicillin and streptomycin (Invitrogen), 5% fetal bovine serum (FBS; Funakoshi, Tokyo, Japan), and 15% horse serum (Gibco, NY, USA). To measure GLP-1 secretion, the cells were plated in 24-well-plates (approximately 1 × 10^5^ cells/well) and cultured for 48 h (17). The cells were treated with octanoate (C8:0), decanoate (C10:0), dodecanoate (C12:0), and embelin (Cayman Chemical, MI, USA) for 1 h. The culture supernatant was collected in the presence of a DPP-IV inhibitor. For siRNA-mediated knockdown, STC-1 cells were transfected with 30 nM siRNA (universal control: 5′-UGGUUUACAUGUCGACUAA-3′, *Gpr84*: 5′-GUUGGGCUAUCGAUACUUU-3′, Dharmacon, Lafayette, CO, USA) using Lipofectamine 2000 transfection reagent (Invitrogen), following the manufacturer's instructions. For inhibitor treatment, STC-1 cells were pretreated with the Gα(i/o) blocker NF023 (10 μM; Calbiochem, CA, USA), Gi/o-type G protein inactivator pertussis toxin (PTX; 1 μg/mL; Wako), Gβγ blocker Gallein (30 μM; Merck Millipore), the phosphoinositide-specific phospholipase C (PLC) inhibitor U73122 (1 μM; Wako), or GPR40 antagonist GW1100 (10 μM; Merck Millipore) for 20 min prior to the addition of decanoate (C10:0).

### HEK293 Cell Culture

Flp-In T-REx HEK293 cells were transfected with a mixture containing mouse HA–*Gpr84* or human FLAG-*GPR40* cDNA in pcDNA5/FRT/TO and pOG44 vectors using Lipofectamine (*Invitrogen*) as described previously (20). Flp-In GPR84 or GPR40 T-REx HEK293 cells were cultured in DMEM containing 10 μg/mL blasticidin S (Funakoshi, Tokyo, Japan), 100 μg/mL hygromycin B (*Invitrogen*), and 10% FBS. The cells were incubated at 37 °C in an atmosphere of 5% CO_2_. To determine cAMP levels, cells were plated on 24-well-plates (~1 × 10^5^ cells/well) and incubated for 24 h. Each well was then treated with or without doxycycline (10 μg/mL) for 24 h. The cells were treated with 3-isobutyl 1-methylxanthine (IBMX; 500 μM; Sigma; phosphodiesterase inhibitor) for 30 min and then stimulated with forskolin (2 μM; Sigma; adenylate cyclase activator) for 10 min. cAMP concentration wa determined using a cAMP enzyme immunoassay (EIA) kit (Cayman Chemical) according to the manufacturer's protocol (20). For intracellular calcium ([Ca^2+^]i) response analysis, cells were plated on 96-well black plates (~3 × 10^4^ cells/well) and incubated for 24 h. Each well was treated with or without doxycycline (10 μg/mL) for 24 h. After treatment, the cells were further incubated in Hank's balanced salt solution (pH 7.4) containing a calcium assay kit component A (Molecular Devices, CA, USA) for 1 h at 37 °C. These plates were placed on a FlexStation 3 multi-mode microplate reader, and the mobilization of [Ca^2+^]i was monitored (17).

### Statistical Analysis

All values are presented as mean ± standard error of mean (SEM). Differences between groups were examined for statistical significance using two-tailed unpaired Student's *t*-test (two groups) and two-tailed one-way analysis of variance, followed by Dunnett's or Tukey-Kramer's post-hoc test (≥ three groups). ROUT and Smirnov-Grubbs' tests were used for evaluating outliers.

## Results

### MCT Intake Improved Metabolic Functions in Mice

**Figure 1:**
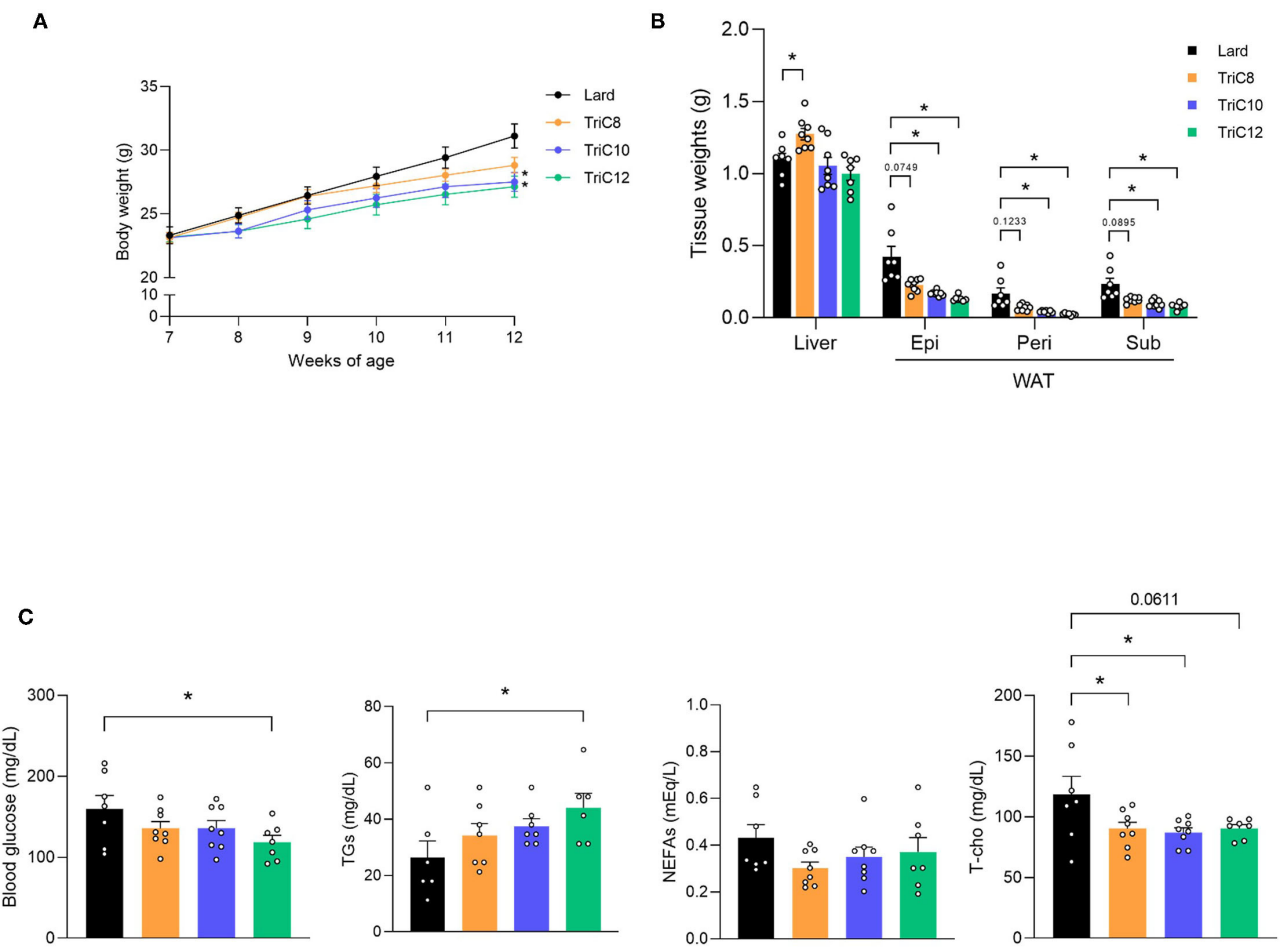
Medium-chain triglyceride (MCT) intakes suppresses body and fat weight increase compared with lard intake. **(A)** Body weight gain for 5 weeks in Lard- or MCT diet-fed mice (*n* = 7–8). **(B)** Tissue weight in Lard or MCT diet-fed mice at 12 weeks of age (*n* = 7–8). **(C)** Blood glucose, plasma triglycerides (TGs), non-esterified fatty acids (NEFAs), and total cholesterol (T-cho) levels in Lard or MCTs diet-fed mice at 12 weeks of age were measured after fasting for 5 h (*n* = 6–8). Dunnett's test; ^*^P < 0.05, compared with Lard-diet. All data are presented as the means ± standard error of mean (SEM). Lard, Lard diet; TriC8, octanoate (C8:0) triglyceride diet; TriC10, decanoate (C10:0) triglyceride diet; TriC12, dodecanoate (C12:0) triglyceride diet. Epi, epididymal adipose tissues; peri, perirenal adipose tissues; sub, subcutaneous adipose tissues; WAT, white adipose tissue.

We first investigated the MCT-induced changes in metabolic parameters after a 20% fat diet, which replaced lard with each type of MCT (octanoate; TriC8, decanoate; TriC10, and dodecanoate; TriC12). After 5 week-feeding of MCT diet (Supplementary Table 1), the body weights of TriC10 and TriC12 diet-fed mice were significantly lower than those of Lard diet-fed mice during growth (Lard > TriC8 > TriC10 > TriC12; Figure 1A). In addition, the weight of white adipose tissue (WAT) was also lower in TriC10 and TriC12 diet-fed mice than in Lard diet-fed mice at 12 weeks of age (Lard > TriC8 > TriC10 > TriC12), whereas the liver weight of TriC8 diet-fed mice was significantly higher than those of Lard diet-fed mice (Figure 1B). The levels of plasma MCFAs C10:0 and C12:0 but not C8:0 were significantly increased by each type of MCT diet (Supplementary Figure 1A), whereas plasma ketone body levels were comparable between Lard and each type of MCT diet-fed mice (Supplementary Figure 1B). Moreover, plasma glucose levels in TriC12 diet-fed mice were significantly lower (Lard > TriC8 > TriC10 > TriC12), and plasma total cholesterol levels in all MCTs diet-fed mice were lower than those in Lard diet-fed mice (Figure 1C). In addition, plasma TG levels in TriC12 diet-fed mice were significantly higher than those in Lard-fed mice (Lard < TriC8 < TriC10 < TriC12). Thus, although all MCT intake prevented obesity, different types of MCTs showed different metabolic functions.

### Oral Administration of MCTs Enhanced Insulin Tolerance in Mice

**Figure 2:**
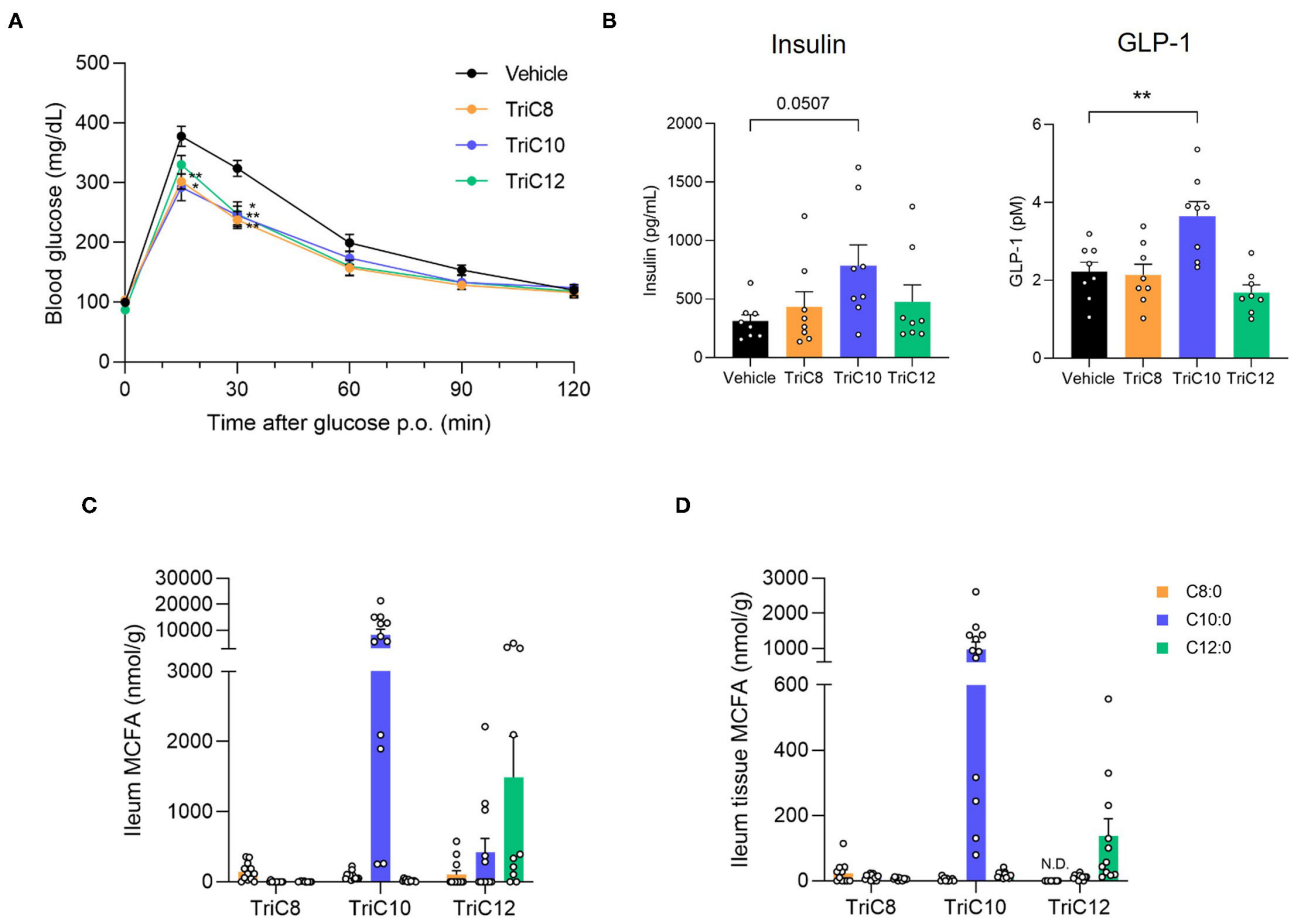
Medium-chain triglyceride (MCT) administration enhances glucose tolerance. **(A)** Oral glucose tolerance test (OGTT) was performed at 1 h post-MCT administration (2.4 g/kg body weight) (*n* = 7). ^**^P < 0.01; ^*^P < 0.05 (Tukey-Kramer test). **(B)** The plasma levels of insulin (left; *n* = 8–10) and GLP-1 (right; *n* = 8–10) at 1.5 h after the oral administration of MCTs. ^**^P < 0.01 vs. vehicle control (Dunnett's test). **(C)** and **(D)** Medium-chain fatty acid (MCFA) levels in ileal content **(C)** or tissue **(D)** of ileum at 1 h after the oral administration of MCTs (*n* = 10–12). The concentration of MCFAs were measured by liquid chromatography/mass spectrometry (LC/MS). TriC8, octanoate (C8:0) triglyceride; TriC10, decanoate (C10:0) triglyceride; TriC12, dodecanoate (C12:0) triglyceride.

We next performed OGTTs by oral MCT administration to examine the influence of MCT intake on glucose homeostasis. Following administration of MCTs (2.4 g/kg) and glucose, we found that the administration of all MCT types individually significantly suppressed the increase in blood glucose level compared to that in control mice (Figure 2A). Moreover, plasma insulin levels following glucose administration at 30 min in all MCT-administered mice were higher than those in control mice, whereas plasma GLP-1 levels in TriC10- but not TriC8- and TriC12-administered mice were significantly higher than those in control mice (Figure 2B). GLP-1 is secreted from enteroendocrine L cells, which mainly exist in the ileum and colon. At 1 h after the oral administration of each type of MCTs, C10:0 was detected in the ileal content and tissue at the highest levels among all MCFAs (Figures 2C,D). Thus, oral administration of MCTs enhanced glucose tolerance and increased plasma GLP-1 levels, especially with TriC10 administration.

### Decanoate and Dodecanoate Promoted GLP-1 Secretion in STC-1 Cells

**Figure 3:**
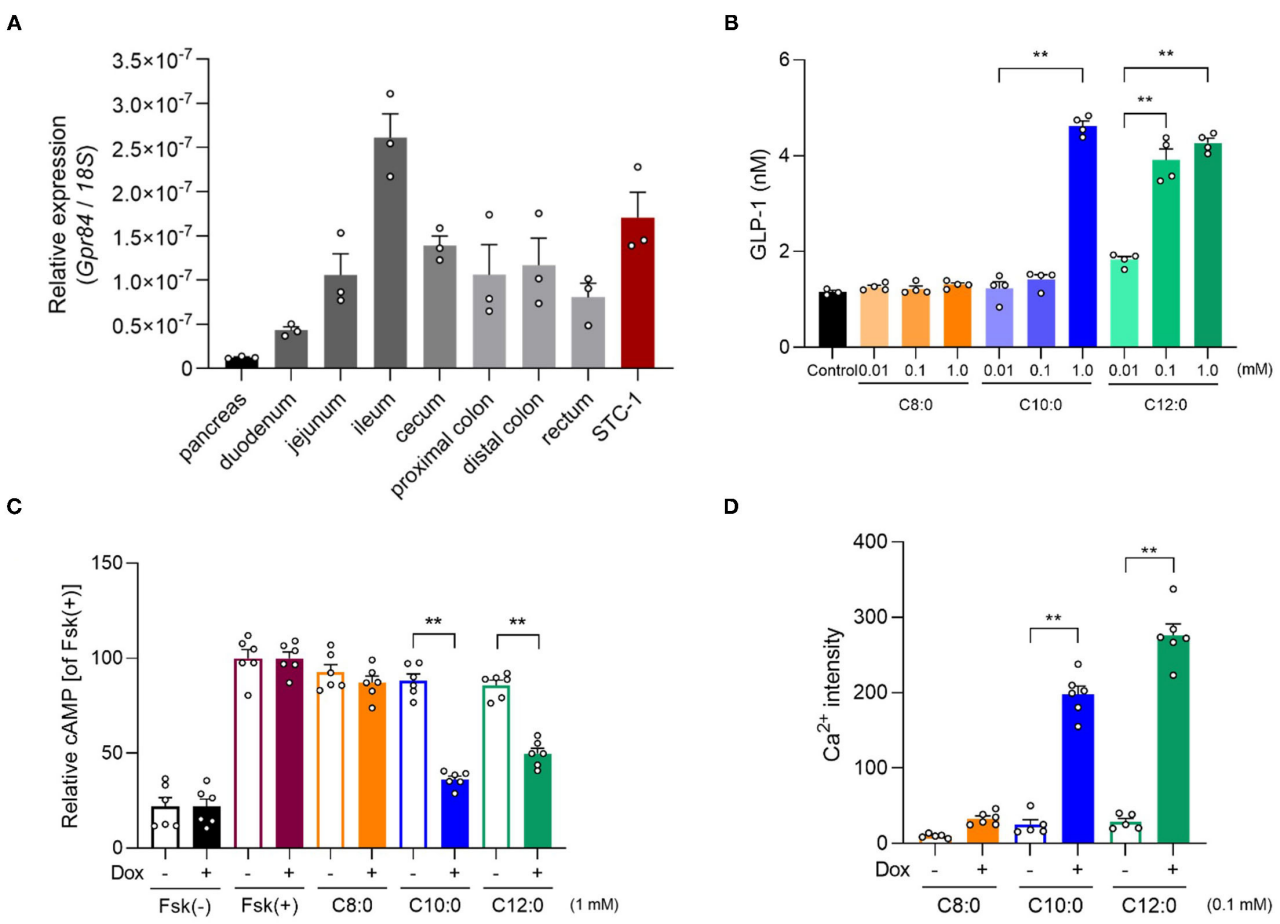
Medium-chain fatty acid (MCFA) decanoate (C10:0) and dodecanoate (C12:0) promotes GLP-1 secretion in STC-1 cells. **(A)** Expression of *Gpr84* in the pancreas, small intestine (duodenum, jejunum, and ileum), cecum, colon (proximal and distal colon), and STC-1 cells (*n* = 3). **(B)** GLP-1 levels in response to MCFAs (0.01, 0.1 or 1 mM, respectively) in STC-1 cells (*n* = 3–4). **(C)** cAMP inhibition assay for MCFAs (1 mM, respectively) using GPR84-expressing HEK293 cells. Cells were cultured for 24 h and then treated with or without 10 μg/mL of doxycycline (Dox, *n* = 6). All data are presented as relative to the forskolin (Fsk)-induced cAMP levels. **(D)** Mobilization of intracellular calcium ([Ca^2+^]i) induced by MCFAs (0.1 mM, respectively) was monitored using GPR40-expressing HEK293 cells. Data are presented as Ca^2+^ intensity. Cells were cultured for 24 h and then treated with or without 10 μg/mL of Dox (*n* = 5–6). ^**^P < 0.01 (Tukey-Kramer test). These experiments were performed using independent cultures from two biological replicates. All data are presented as the mean ± standard error of mean (SEM).

MCFAs are ligands for FFAR GPR84. Hence, we examined the influence of GPR84 on GLP-1 secretion by MCTs. *Gpr84* was expressed in the intestine, especially the ileum, and STC-1 cells as enteroendocrine cells, but not in the pancreas (Figure 3A). C10:0 and especially C12:0 strongly induced GLP-1 secretion in a dose-dependent manner in STC-1 cells, whereas C8:0 hardly induced these effects (Figure 3B). GPR40 is also a receptor for MCFAs; GPR84 couples to Gi/o, whereas GPR40 couples to Gq (1). Using a heterologous expression system, we found that C10:0 and C12:0 but not C8:0 significantly suppressed cAMP levels induced by forskolin in GPR84-overexpressing HEK293 cells (C10:0 > C12:0), whereas C10:0 and C12:0 but not C8:0 significantly increased [Ca^2+^]i levels in GPR40-overexpressing HEK293 cells (C10:0 < C12:0), and these effects were not observed in doxycycline (–) control HEK293 cells (Figures 3C,D). Thus, C10:0 and C12:0 but not C8:0 promotes GLP-1 secretion in STC-1 cells.

### Decanoate Promoted GLP-1 Secretion *via* GPR84 in STC-1 Cells

**Figure 4:**
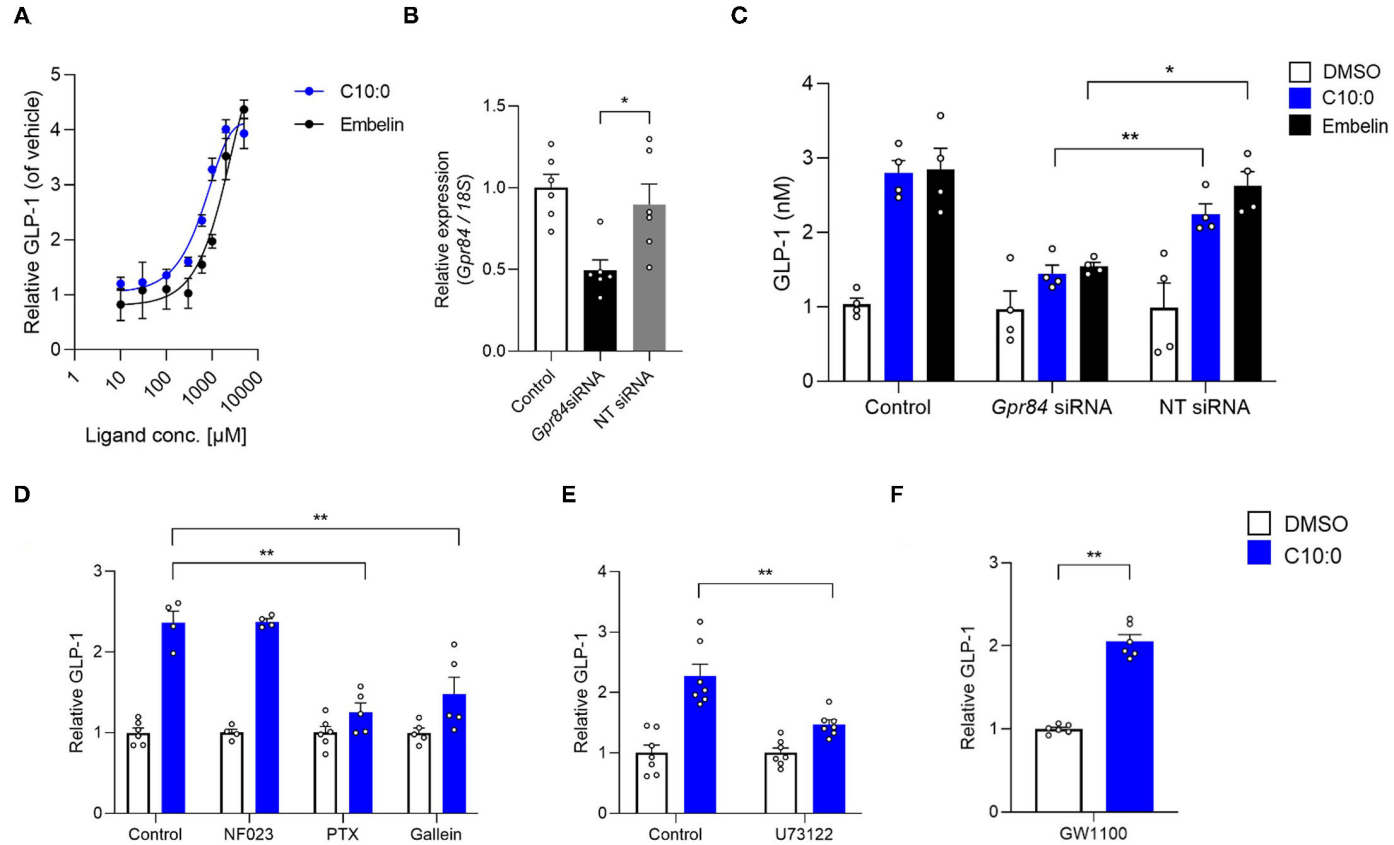
Medium-chain fatty acid (MCFA) decanoate (C10:0) promotes GLP-1 secretion via GPR84 in STC-1 cells. **(A)** STC-1 cells were treated with various doses of C10:0 and GPR84 agonist embelin (0.01, 0.03, 0.1, 0.3, 0.6, 1, 2, and 5 mM). GLP-1 concentration in the culture medium was measured (*n* = 6). Data are represented as relative to the GLP-1 protein levels in non-stimulated cells. **(B)** RNAi efficiency by *Gpr84* siRNA (*n* = 6). *Gpr84* expression is represented as relative to its expression in the untreated control cells. **(C)** Inhibitory effects of *Gpr84* siRNA on GLP-1 secretion in cells treated with C10:0 (1 mM) and embelin (1 mM; *n* = 4). **(D–F)** Cells were stimulated with C10:0 (500 μM) after pretreatment with NF023 (10 μM), PTX (1 μg/mL), Gallein (30 μM), U73122 (1 μM), or GW1100 (10 μM) for 20 min (*n* = 4–6). Data are represented as relative to the GLP-1 protein levels in C10:0 non-stimulated cells. ^**^P < 0.01; ^*^P < 0.05 (Tukey-Kramer test). These experiments were performed using independent cultures from two biological replicates. All data are presented as the mean ± standard error of mean (SEM).

Moreover, we investigated whether C10:0-stimulated GLP-1 secretion is mediated by GPR84. The GPR84 agonist embelin (21), as well as C10:0, directly promoted GLP-1 secretion in a dose-dependent manner in STC-1 cells (Figure 4A). The GLP-1 secretion by either C10:0 or embelin stimulation was significantly inhibited by *Gpr84* RNA interference in STC-1 cells (Figures 4B,C). Additionally, C10:0-stimulated GLP-1 secretion was effectively inhibited by treatment with pertussis toxin (PTX) (Gi/o-type G protein inactivator) (18, 22), Gallein (Gβγ blocker) (18, 23), and U73122 (PLC inhibitor), but not NF023 (Gα(i/o) blocker) (18, 24) (Figures 4D,E) in STC-1 cells. These results showed that C10:0-stimulated GLP-1 secretion in STC-1 cells was mediated by G(i/o)βγ-PLC signaling, but not Gq signaling. Moreover, C10:0-stimulated GLP-1 secretion in STC-1 cells was not abolished by treatment with the GPR40 antagonist GW1100 (25) (Figure 4F). Thus, C10:0-stimulated GLP-1 secretion is promoted via GPR84 rather than via GPR40.

### Decanoate Enhanced Glucose Tolerance *via* GPR84-Mediated GLP-1 Secretion in Mice

**Figure 5:**
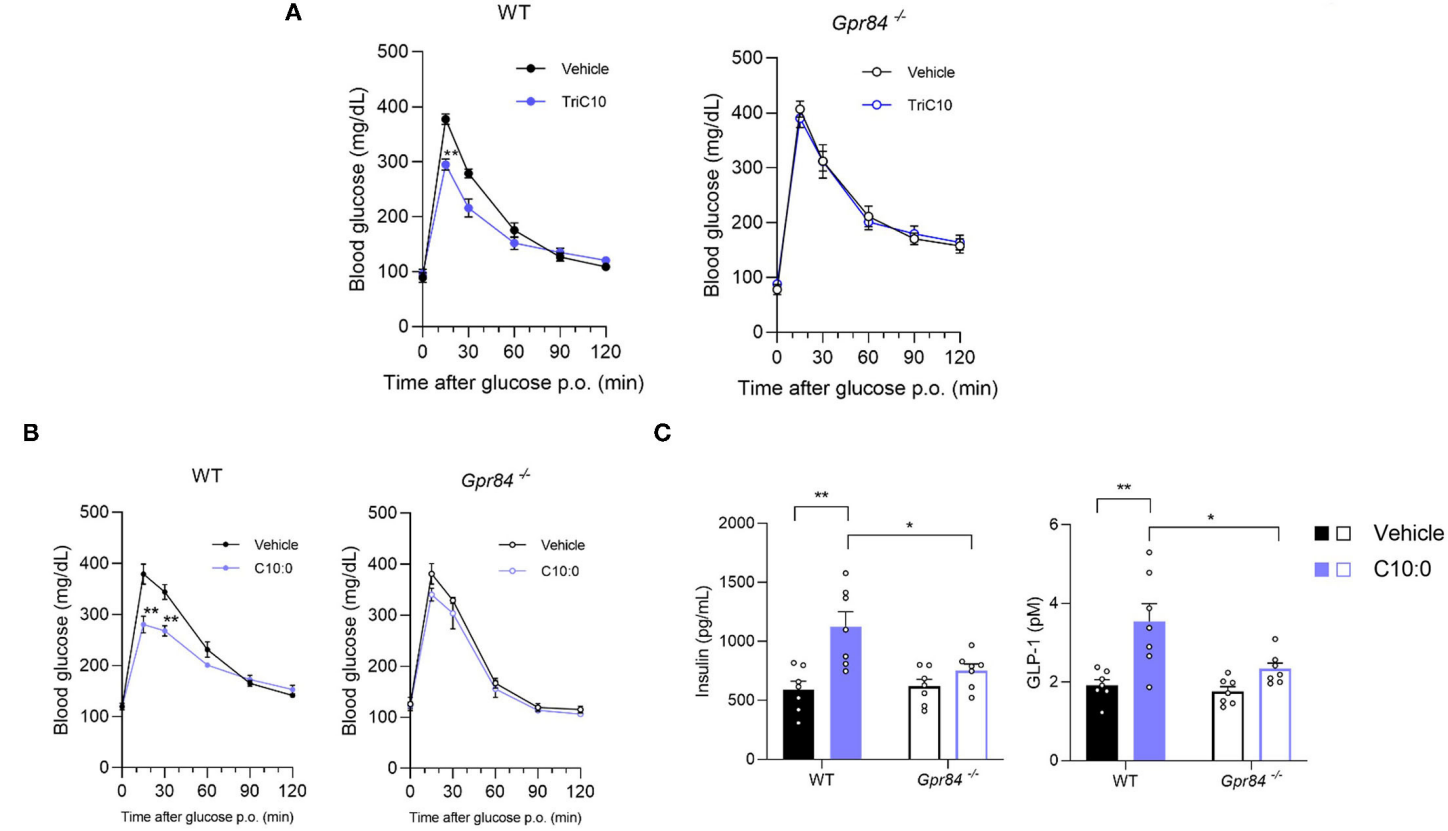
Decanoate (C10:0) enhanced glucose tolerance via GPR84-mediated GLP-1 secretion in mice. **(A)** Oral glucose tolerance test (OGTT) was performed at 1 h post-decanoate triglyceride administration (2.4 g/kg body weight) (left, WT mice; *n* = 7, 6; right, *Gpr84*^−/−^ mice; *n* = 5). **(B)** OGTT was performed at 2 h post-decanoate administration (1 g/kg body weight) (left, WT; right, *Gpr84*^−/−^ mice; *n* = 7). **(C)** The plasma levels of insulin (left) and GLP-1 (right) at 2.5 h after the oral administration of decanoate (*n* = 7). ^**^P < 0.01, ^*^P < 0.05 (Tukey-Kramer test). All data are presented as the means ± standard error of mean (SEM).

We next investigated the effects of C10:0 via GPR84 on glucose homeostasis and GLP-1 secretion *in vivo*. In OGTT, either TriC10 or C10:0 administration significantly suppressed the increase in blood glucose levels in wild-type mice, whereas this effect was attenuated in *Gpr84*^−/−^ mice (Figures 5A,B). Additionally, oral C10:0 administration markedly increased plasma GLP-1 and insulin levels in wild-type mice, whereas these effects were attenuated in *Gpr84*^−/−^ mice (Figure 5C). Thus, C10:0 administration enhances glucose tolerance via GPR84-mediated GLP-1 secretion.

### Decanoate Supplementation Improved Metabolic Functions in Obese Mice

**Figure 6:**
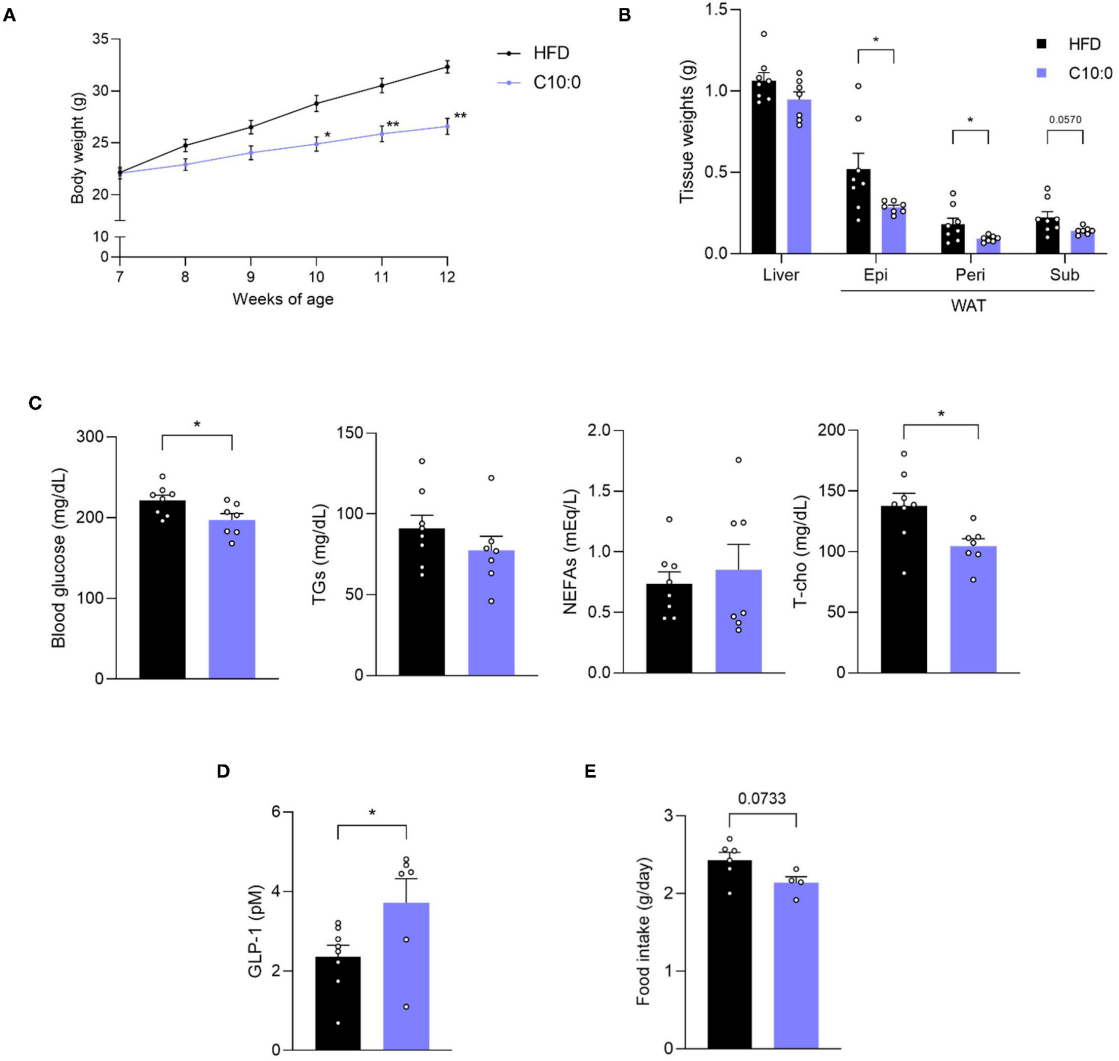
Decanoate (C10:0) intake improves metabolic functions in high-fat diet (HFD)-induced obese model mice. **(A)** Body weight gain for 5 weeks in HFD- or C10:0-supplemented HFD-fed mice (n = 8, 7) and **(B)** tissue weight in HFD- or C10:0-supplemented HFD-fed mice at 12 weeks of age (n = 8, 7). **(C)** Blood glucose, plasma triglycerides (TGs), non-esterified fatty acids (NEFAs), and total cholesterol (T-cho) levels in HFD- or C10:0-supplemented HFD-fed mice at 12 weeks of age (n = 8, 7), **(D)** plasma GLP-1 levels (n = 8, 6) in HFD- or C10-supplemented HFD-fed mice at 12 weeks of age were measured after fasting for 5 h. **(E)** Daily food intake measured at 7 weeks of age (n = 6, 4) ^**^P < 0.01, ^*^P < 0.05 (**A**, Tukey-Kramer test, **B**–**E**, Student's t test). All data are presented as the means ± standard error of mean (SEM). C10:0, 5% decanoate (C10:0)-supplemented HFD. Epi, epididymal adipose tissues; peri, perirenal adipose tissues; sub, subcutaneous adipose tissues; WAT, white adipose tissue.

Finally, we investigated the beneficial effects of C10:0 supplementation on the metabolic functions of HFD-induced obese mice. After C10:0-supplemented HFD-feeding (Supplementary Table 2), we observed that the body weights of the mice were markedly lower than those of HFD-fed control mice during growth (Figure 6A). In addition, the weight of WAT was also lower in C10:0-supplemented HFD-fed mice than in HFD-fed control mice at 12 weeks of age (Figure 6B). Plasma C10:0 levels were significantly increased by C10:0-supplemented HFD feeding (Supplementary Figure 2). Moreover, plasma glucose and total cholesterol levels in C10:0-supplemented HFD-fed mice were significantly lower than those in HFD-fed control mice (Figure 6C). Plasma GLP-1 levels in C10:0-supplemented HFD-fed mice were significantly higher than those in HFD-fed control mice (Figure 6D). Additionally, food intake in C10:0-supplemented HFD-fed mice tended to be lower than that in the HFD-fed control mice (Figure 6E). Thus, C10:0 supplementation efficiently prevented HFD-induced obesity.

## Discussion

MCT intake exhibits various beneficial metabolic effects. However, the individual functional differences between MCT types remain unclear. In this study, we found that decanoate is clearly functionable to regulate glucose homeostasis by GLP-1 secretion via GPR84, improves insulin sensitivity, and prevents obesity.

All MCT intakes showed improvements in obesity and metabolic parameters. However, the beneficial metabolic effects of each type of MCT showed some differences. Although TriC12 exhibited the highest anti-obesity effect and hyperglycemia suppression among the different types of MCT, it increased the levels of plasma TGs. On the other hand, although TriC8 showed a weaker anti-obesity effect than TriC10 and TriC12, it exhibited the lowest plasma NEFA levels among the different MCT types. Importantly, TriC8 intake markedly increased liver weight. Dietary water-soluble nutrients are carried to the liver from the intestine. These results suggest that C8:0 is directly metabolized in the liver and released from the portal vein due to its highest water solubility among MCFAs (4, 5), and thereby, hardly influences the circulating and peripheral plasma lipids. C8:0 does not activate GPR84 and GPR40 (1, 11). Therefore, although the effect of TriC8 intake on liver weight does not depend on GPR84, other C8:0-specific receptors or C8:0 metabolism in the liver may be related to an increase in liver weight.

In STC-1 cells, C12:0 stimulation strongly promoted GLP-1 secretion compared to C10:0 stimulation. However, in the mice, oral administration of TriC10 increased plasma GLP-1 levels rather than TriC8 and TriC12. This difference between *in vivo* and *in vitro* experiments may cause the fact that C10:0 could reach to distal intestine, where GLP-1-positive enteroendocrine L cells are localized (14, 15), after TriC10 administration, whereas C12:0 was hardly detected in the ileum. Hence, these results indicate that it is important to understand the biological functions and the physiological dynamics of individual MCTs and MCFAs.

Additionally, MCFAs act as ligands not only for GPR84, but also for GPR40. However, GPR40 is also a receptor for LCFAs (1). Hence, under HFD-fed conditions, activation of GPR40 by TriC10 and TriC12 intake may hardly occur because LCFA levels are much higher than C10:0 and C12:0 levels in the plasma and intestine (17). Moreover, our data showed that C10:0-induced GLP-1 secretion was sufficiently suppressed by *Gpr84* siRNA and inhibition of Gi/o signaling, but not Gq signaling, which was related to GPR40 in STC-1 cells; further, C10:0-induced GLP-1 secretion was not abolished by treatment with a GPR40 antagonist. Additionally, GPR84 deficiency sufficiently attenuated the enhancement of glucose tolerance, and increased plasma GLP-1 and insulin levels following either TriC10 or C10:0 administration. However, we cannot completely deny the influence of GPR40 on beneficial metabolic effects and GLP-1 secretion by each MCFA.

Moreover, GPR84 may also have beneficial metabolic effects via the secretion of other gut hormones. We showed that *Gpr84* mRNA was sufficiently expressed in the enteroendocrine cell line STC-1, and *Gpr84* mRNA expression was the highest in the ileum, where GLP-1 and/or anorectic gut hormone peptide (PYY)-positive enteroendocrine L cells are localized (14, 15). MCT stimulates the secretion of the PYY in the distal intestine but not the proximal intestine (26) and, to a lesser extent, the secretion of another incretin glucose-dependent insulinotropic polypeptide (GIP), which is secreted from K cells, are localized in the proximal intestine (27). On the other hand, we cannot also deny the influence of indirect GLP-1 secretion via C10:0-stimulated GPR84 activation by neuronal or immune cells. For example, vagal afferent activation indirectly promotes GLP-1 secretion (28), and GPR40 stimulation also promotes GLP-1 secretion via the afferent vagal nerve (29). Further studies using tissue-specific GPR84-deficient mice, or GPR40 and GPR40/GPR84 double-deficient mice are needed to clarify these problems.

## Conclusions

Our data showed that TriC10 intake, administration of TriC10 and C10:0, and C10:0 supplementation exerted inhibitory effects on HFD-induced obesity and improved glucose homeostasis by GPR84-mediated GLP-1 secretion in *in vivo* and *in vitro* studies. Our results may contribute to the development of functional foods and oils using MCTs, especially TriC10, for the prevention of metabolic disorders, such as obesity and type 2 diabetes.

## Data Availability Statement

The original contributions presented in the study are included in the article/Supplementary Material, further inquiries can be directed to the corresponding author/s.

## Ethics Statement

The animal study was reviewed and approved by the Committee on the Ethics of Animal Experiments of the Kyoto University Animal Experimentation Committee (Lif-K21020) and the Tokyo University of Agriculture and Technology (permit number: 28–87).

## Author Contributions

HN performed the experiments, interpreted data, and wrote the paper. RO-K performed the experiments, interpreted data, and wrote the paper. YM interpreted data and wrote the paper. MI interpreted data and wrote the paper. IK supervised the project, interpreted data, wrote the paper, and had primary responsibility for the final content. All authors read and approved the final manuscript.

## Funding

This work was partly supported by research grants from the JSPS KAKENHI (JP21H04862), JST-OPERA (JPMJOP1833), JST-Moonshot R&D (JPMJMS2023), and Nisshin OilliO Group, Ltd (to IK). This study received funding from Nisshin OilliO Group, Ltd. The funder was not involved in the study design, collection, analysis, interpretation of data, the writing of this article or the decision to submit it for publication.

## Conflict of Interest

The authors declare that the research was conducted in the absence of any commercial or financial relationships that could be construed as a potential conflict of interest.

## Publisher's Note

All claims expressed in this article are solely those of the authors and do not necessarily represent those of their affiliated organizations, or those of the publisher, the editors and the reviewers. Any product that may be evaluated in this article, or claim that may be made by its manufacturer, is not guaranteed or endorsed by the publisher.

## Supplementary Material

Supplementary Figure 1 Plasma medium-chain fatty acids (MCFAs) and ketone body levels under medium-chain triglycerides (MCTs) diet-fed condition. **(A)** MCFAs (C8:0, C10:0, and C12:0) levels (n = 5–8) and **(B)** ketone body levels (n = 5–6) in the plasma of Lard or MCT diet-fed mice for 5 weeks. Samples collected after fasting for 5 h were measured by liquid chromatography/mass spectrometry (LC/MS). Dunnett's test; ^**^P < 0.01, compared with Lard-diet. All data are presented as the mean ± standard error of mean (SEM). Lard, Lard diet; TriC8, octanoate (C8:0) triglyceride diet; TriC10, decanoate (C10:0) triglyceride diet; TriC12, dodecanoate (C12:0) triglyceride diet.

Supplementary Figure 2 Plasma C10:0 levels under C10:0-supplemented high-fat diet (HFD)-fed condition. C10:0 levels in the plasma of HFD- or 5% C10:0-supplemented HFD-fed mice for 5 weeks (n = 8, 7). Samples which collected after fasting for 5 h were measured by liquid chromatography/mass spectrometry (LC/MS). Student's t test; ^**^P < 0.01. All data are presented as the mean ± standard error of mean (SEM). C10:0, 5% decanoate (C10:0)-supplemented HFD.

Supplementary Table 1 Diet composition in medium-chain triglyceride (MCT) intake experiments. Lard, Lard diet; TriC8, octanoate (C8:0) triglyceride diet; TriC10, decanoate (C10:0) triglyceride diet; TriC12, dodecanoate (C12:0) triglyceride diet.

Supplementary Table 2 Diet composition in decanoate (C10:0) supplementation experiments. HFD, high-fat diet; C10:0, 5% decanoate (C10:0)-supplemented HFD.
