# Supplementary figures and images for: Dietary Medium-Chain Triglyceride Decanoate Affects Glucose Homeostasis Through GPR84-Mediated GLP-1 Secretion in Mice
Source: Front Nutr. 2022 Mar 24;9:848450. doi: 10.3389/fnut.2022.848450 (PMC8987919; doi:10.3389/fnut.2022.848450)

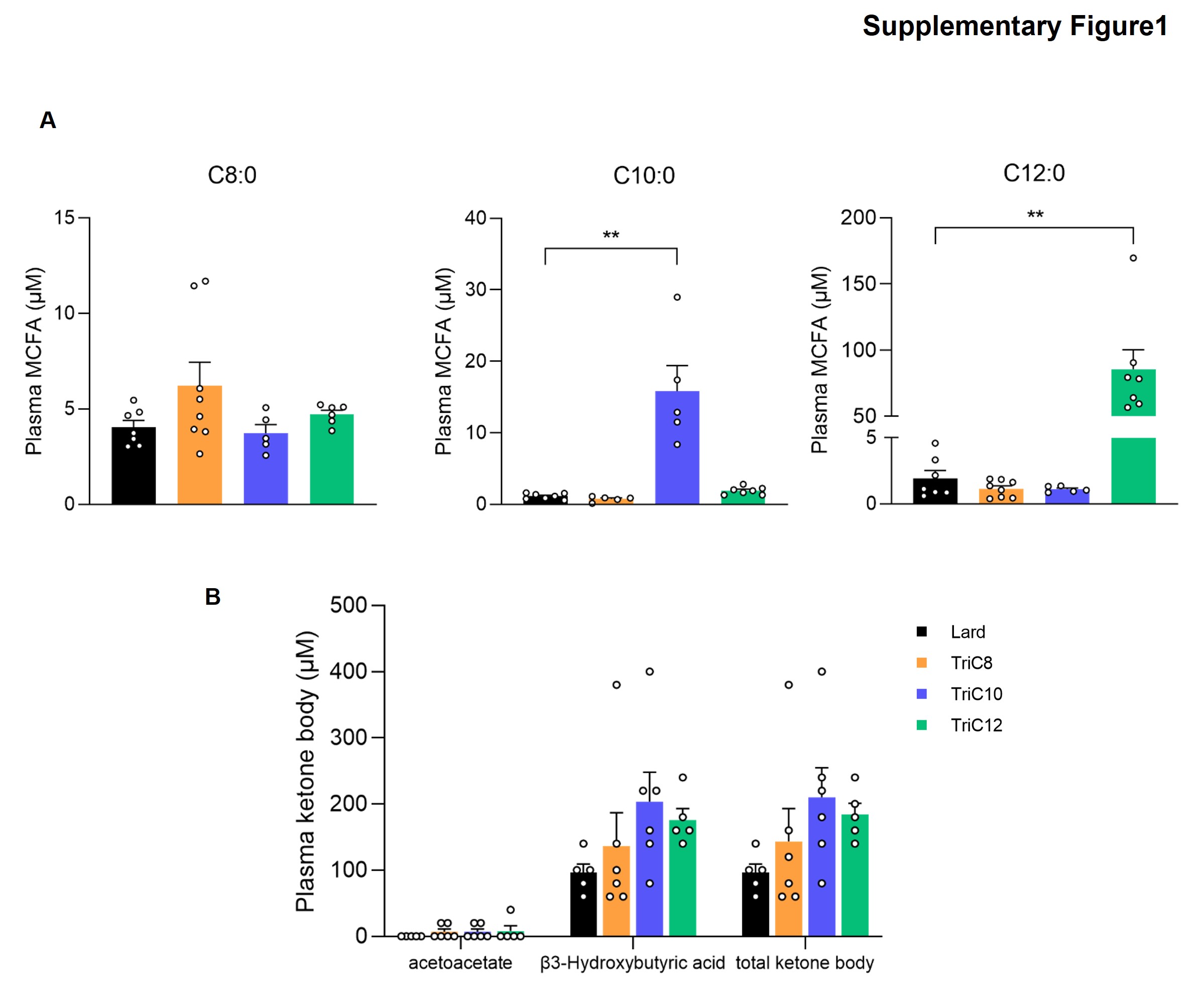

Supplement: Supplementary Figure 1 — Plasma medium-chain fatty acids (MCFAs) and ketone body levels under medium-chain triglycerides (MCTs) diet-fed condition. (A) MCFAs (C8:0, C10:0, and C12:0) levels (n = 5–8) and (B) ketone body levels (n = 5–6) in the plasma of Lard or MCT diet-fed mice for 5 weeks. Samples collected after fasting for 5 h were measured by liquid chromatography/mass spectrometry (LC/MS). Dunnett's test; **P < 0.01, compared with Lard-diet. All data are presented as the mean ± standard error of mean (SEM). Lard, Lard diet; TriC8, octanoate (C8:0) triglyceride diet; TriC10, decanoate (C10:0) triglyceride diet; TriC12, dodecanoate (C12:0) triglyceride diet. [file Image_1.JPEG]

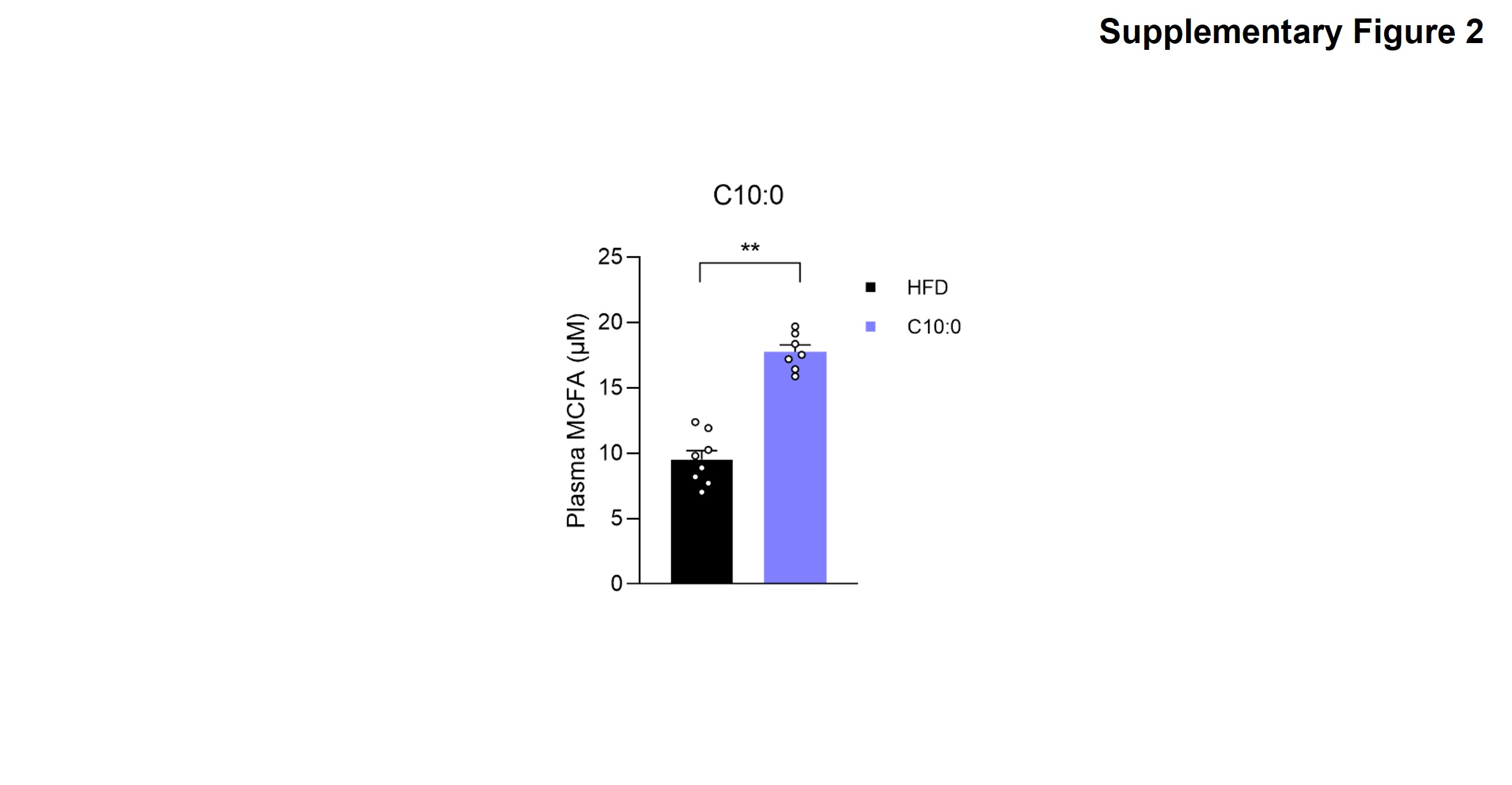

Supplement: Supplementary Figure 2 — Plasma C10:0 levels under C10:0-supplemented high-fat diet (HFD)-fed condition. C10:0 levels in the plasma of HFD- or 5% C10:0-supplemented HFD-fed mice for 5 weeks (n = 8, 7). Samples which collected after fasting for 5 h were measured by liquid chromatography/mass spectrometry (LC/MS). Student's t test; **P < 0.01. All data are presented as the mean ± standard error of mean (SEM). C10:0, 5% decanoate (C10:0)-supplemented HFD. [file Image_2.jpg]
