# Supplementary material for: Dietary Medium-Chain Triglyceride Decanoate Affects Glucose Homeostasis Through GPR84-Mediated GLP-1 Secretion in Mice
Source: Front Nutr. 2022 Mar 24;9:848450. doi: 10.3389/fnut.2022.848450 (PMC8987919; doi:10.3389/fnut.2022.848450)
Supplement: Supplementary Table 1 — Diet composition in medium-chain triglyceride (MCT) intake experiments. Lard, Lard diet; TriC8, octanoate (C8:0) triglyceride diet; TriC10, decanoate (C10:0) triglyceride diet; TriC12, dodecanoate (C12:0) triglyceride diet. [file Table_1.doc]

**Supplementary Table 1.** Diet composition in medium-chain triglyceride (MCT) intake experiments. Lard, Lard diet; TriC8, octanoate (C8:0) triglyceride diet; TriC10, decanoate (C10:0) triglyceride diet; TriC12, dodecanoate (C12:0) triglyceride diet.

|  |  |  |  |  |
| --- | --- | --- | --- | --- |
| Ingredient | Control | MCT | | |
|  | Lard | TriC8 | TriC10 | TriC12 |
|  | gm(%) | | | |
| Casein | 20.000 | 20.000 | 20.000 | 20.000 |
| Cystine | 0.300 | 0.300 | 0.300 | 0.300 |
| β-Corn Starch | 26.746 | 26.746 | 26.746 | 26.746 |
| α-Corn Starch | 13.200 | 13.200 | 13.200 | 13.200 |
| Sucrose | 10.000 | 10.000 | 10.000 | 10.000 |
| Cellulose Powder | 5.000 | 5.000 | 5.000 | 5.000 |
| Soybean Oil | 2.000 | 2.000 | 2.000 | 2.000 |
| Lard | 18.000 | - | - | - |
| TriC8 | - | 18.000 | - | - |
| TriC10 | - | - | 18.000 | - |
| TriC12 | - | - | - | 18.000 |
| AIN-93G Mineral Mix | 3.500 | 3.500 | 3.500 | 3.500 |
| AIN-93 Vitamin Mix | 1.000 | 1.000 | 1.000 | 1.000 |
| Choline Bitartrate | 0.250 | 0.250 | 0.250 | 0.250 |
| tert-Butylhydroquinone | 0.004 | 0.004 | 0.004 | 0.004 |
| Total | 100.000 | 100.000 | 100.000 | 100.000 |
|  |  |  |  |  |
