# Supplementary material for: Dietary Medium-Chain Triglyceride Decanoate Affects Glucose Homeostasis Through GPR84-Mediated GLP-1 Secretion in Mice
Source: Front Nutr. 2022 Mar 24;9:848450. doi: 10.3389/fnut.2022.848450 (PMC8987919; doi:10.3389/fnut.2022.848450)
Supplement: Supplementary Table 2 — Diet composition in decanoate (C10:0) supplementation experiments. HFD, high-fat diet; C10:0, 5% decanoate (C10:0)-supplemented HFD. [file Table_2.doc]

**Supplementary Table 2.** Diet composition in decanoate (C10:0) supplementation experiments. HFD, high-fat diet; C10:0, 5% decanoate (C10:0)-supplemented HFD.

|  |  |  |
| --- | --- | --- |
| Ingredient | HFD | C10:0 |
|  | gm(%) | |
| Casein, 30 Mesh | 25.845 | 24.553 |
| L-Cystine | 0.388 | 0.368 |
| Maltodextrin 10 | 16.153 | 15.345 |
| Sucrose | 8.891 | 8.446 |
| Cellulose, BW200 | 6.461 | 6.138 |
| Soybean Oil | 3.231 | 3.069 |
| Lard | 31.660 | 30.077 |
| C10:0 | - | 5.000 |
| Mineral Mix S10026 | 1.292 | 1.228 |
| DiCalcium Phosphate | 1.680 | 1.596 |
| Calcium Carbonate | 0.711 | 0.675 |
| Potassium Citrate, 1 H_2_O | 2.132 | 2.026 |
| Vitamin Mix V10001 | 1.292 | 1.228 |
| Choline Bitartrate | 0.258 | 0.245 |
| FD&C Blue Dye #1 | 0.006 | 0.006 |
| Total | 100.00 | 100.00 |
|  |  |  |
